# X-ray Target Shielding and Leakage Radiation Inside the Treatment Capsule of the Zap-X

**DOI:** 10.7759/cureus.31490

**Published:** 2022-11-14

**Authors:** Georg A Weidlich, Justin Keener

**Affiliations:** 1 Radiation Oncology, National Medical Physics & Dosimetry Company, Inc., Palo Alto, USA; 2 Radiation Oncology, Adventist Health System, Littleton, USA

**Keywords:** target plane, patient plane, leakage radiation, x-ray target shielding, zap-x

## Abstract

The ZAP-X represents the first-of-its-kind "self-shielded" therapeutic radiation device, which by novelty, challenges regulators to accommodate it within the existing regulatory framework for radiation protection. To facilitate informed regulatory interpretation, X-ray radiation leakage from the ZAP-X was measured inside the shielded treatment capsule at the level of the patient and X-ray target plane. Measurements were performed on a clinically commissioned system calibrated for reference conditions to deliver 1cGy/MU. Radiation was measured with a FLUKE 451 survey meter and a RadCal ionization chamber as both exposure and dose and presented as a percentage of the system reference dose. Measurements were taken at thirteen locations, eight in the patient plane and five in the X-ray target plane. The results showed a maximum X-ray leakage of 0.000986% in the patient plane and 0.000907% in the target plane. These results are 30 - 100 times lower than existing recommendations as referenced by IEC guidelines standard 60601-2-1 (2020) for radiotherapy linear accelerators (LINACs). Although most conventional LINACs apply a safety factor of 2-5 to the design of collimator shielding and patient dose sparing, the ZAP-X delivers less than 10% of the patient whole body dose compared to this standard, originating from the X-ray target. Even though the ZAP-X intensity modulated radiation therapy (IMRT) factor is significantly higher than conventional Linacs, the absolute dose originating from leakage radiation remains lower by 25. The amount of unintended dose received by the patient's body distant from the isocenter is of interest from the perspective of both clinical and radiation safety. As this whole-body dose is decreased, the resulting treatment-related cancer incidence and mortality rates are decreased accordingly.

## Introduction

The Zap-X is a recently designed and developed, dedicated self-contained and self-shielded radiosurgery system developed and manufactured by ZAP Surgical Systems, Inc. of San Carlos, California. This device is intended for stereotactic radiosurgery (SRS) treatment of benign and malignant intracranial and cervical spine lesions. A 3.0 megavolt (MV) S-band linear accelerator (linac) is the source of therapeutic radiation. Akin to a large gyroscope, the linac is mounted within a combination of yoked gimbals with attached radiation shielding, each of which accurately rotates around a common isocenter. This mechanical construct enables the linac beam to potentially crossfire from approximately 2π steradians of solid angle, as is ideally required for cranial SRS [1].

Accurate therapeutic beam positioning is accomplished through the dual axes, independent accelerator rotations, and precise robotic patient table movements. Most components needed to produce the beam, such as the radiofrequency power source, waveguiding system, beam triggering electronics, and significant radiation shielding, are mounted on or integrated into the rotating spherical patient treatment chamber. The patient is supported on a moveable treatment table that extends outside the iron sphere and is enclosed by additional radiation shielding during radiosurgery. This table shielding consists of a rotary iron shell and a pneumatically raised door on a steel frame.

Due to the ever-increasing IMRT factor of modern precision Radiotherapy systems and the consequent increase in Monitor Units (MU) to deliver such treatments, the dose delivered to the patient's whole body due to target leakage is expected to increase for a given prescription dose to the target. In-patient scatter radiation produced by treatment radiation, and the X-ray target leakage dose contributes to the patient's whole body dose. While the former is unavoidable and a function of energy, field size, and dose delivered, the latter originates directly from the X-ray target and is heavily dependent on the amount of shielding in the collimation system; the exact design of the collimator head directly determines the overall patient whole body dose. In the case of the ZAP-X, 10.4cm to 15.0 cm of Tungsten shielding is used, translating into 3.5 to 5.0 Tenth-Value Layers (TVL) [2]. This is significantly more shielding than used in conventional radiotherapy LINACs. To corroborate the above design and measure the radiation dose delivered to patients, exposure and dose from X-ray leakage were acquired at various positions inside the ZAP-X treatment capsule. The results were expressed with the total dose delivered by the treatment beams.

## Technical report

**Figure 1:**
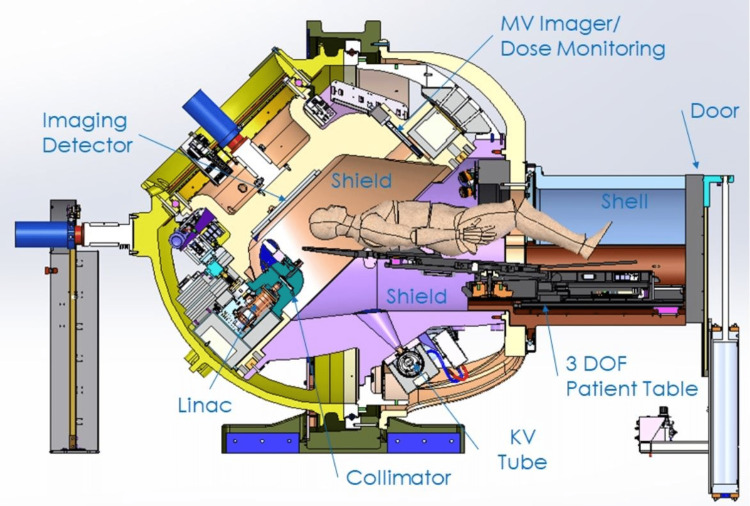
Cross-sectional view of Zap-X MV: megavoltage: DOF: degree of freedom; KV: kilovoltage The author created the image for the system design.

The Zap-X is shown in Figure 1 and has been described in more detail in previous publications [1-2].

**Figure 2:**
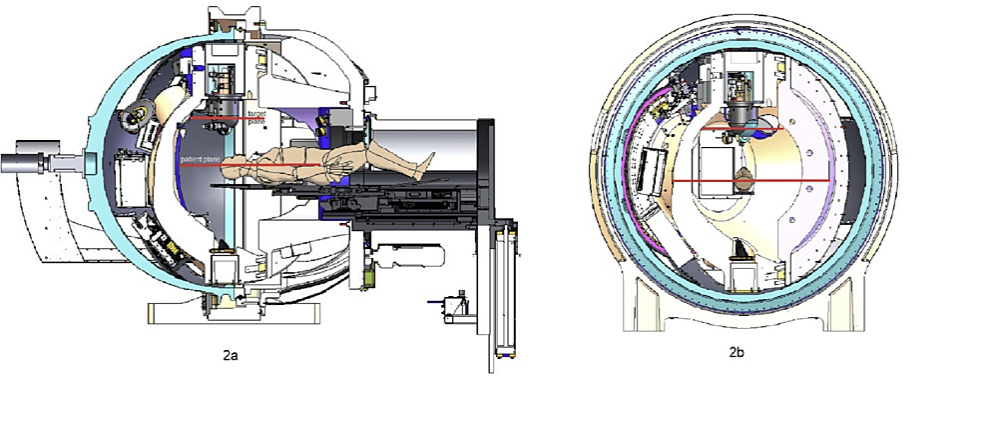
a: Elevation view and b: Cross-sectional view of the patient plane and target plane; measurement planes are indicated in red The author created the image for the experiment design.

**Figure 3:**
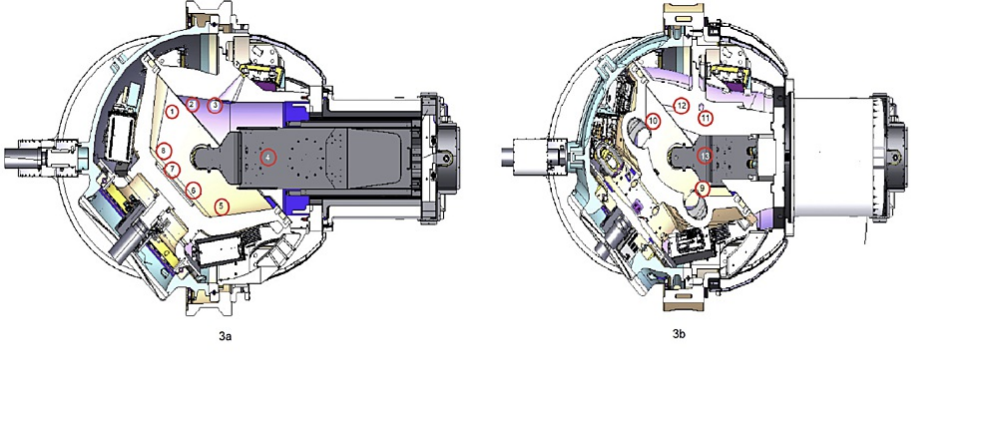
a: Indication of measurement stations in the patient plane; b: Indication of measurement stations in the target plane. The author created the image for the experiment design.

X-ray target leakage radiation was measured in the patient plane at a Source-to-Axis distance (SAD) of 45cm, perpendicular to the beam's central axis (CAX), including the isocenter and the target plane perpendicular to the beam CAX, including the target. All measurements were performed with the beam in the vertical orientation pointing down. A total of thirteen measurement points - eight in the patient plane and five in the target plane were used at a distance of 1 m from the CAX or the largest available distance inside the treatment capsule. The number and location of measurement points were chosen to distribute them equidistantly (30cm) from each other on accessible inside surfaces of the gyroscopically moving treatment capsule. The orientations of the measurement planes are shown in Figures 2a, 2b, and the positions of the measured points are shown in Figures 3a, 3b.

**Figure 4:**
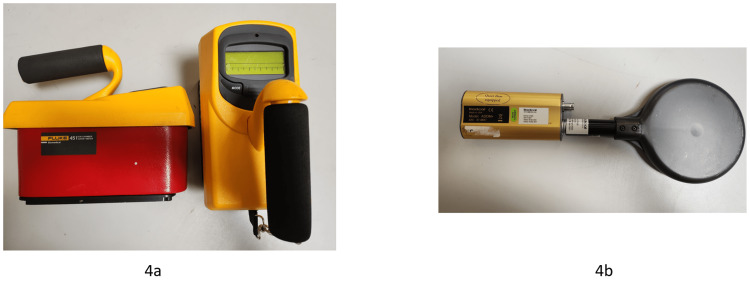
a: Fluke 451 survey meter and b: RadCal leakage ionization chamber

A radiation survey meter (Fluke Biomedical Model 451 BRYR, S/N 0000003284, Fluke Biomedical, Everett, WA) and a large-volume leakage chamber from Radcal (model #ADDM+, S/N 47-0657, Radcal Corporation, Monrovia, CA) were used to measure radiation leakage and are shown in Figure 4a, 4b. Both radiation detectors are calibrated periodically at one-year intervals, and the latest calibration for both systems was less than 12 months ago. Reported readings were acquired with this survey meter; redundant readings were performed with additional survey meters of the same type for verification. All measurements were performed with a blank collimator selected. The collimator wheel was positioned, so the beam intercepted the solid tungsten material; therefore, only X-ray target leakage was detected [2]. For each reading, 1,000 MU were delivered. 1,000 MU corresponds to 1,000 cGy under reference conditions (SAD=45cm, 25mm collimator, dmax=7mm). 1000 cGy was chosen as a typical and most commonly used dose per fraction with the ZAP-X.

**Figure 5:**
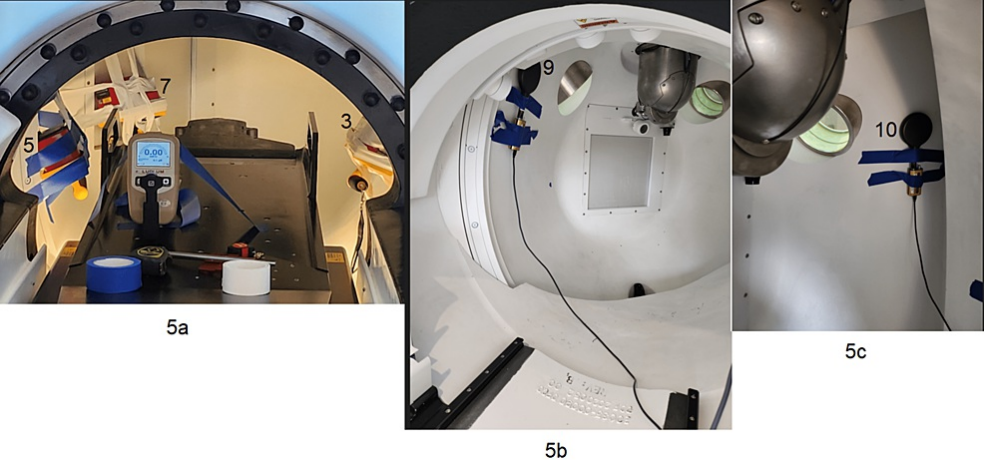
a: Measurement positions 3, 5, 7; b: position 9; c: position 10 All positions were measured with a FLUKE survey meter and the RadCal ionization chamber. Author acquired image.

Measurements setup at positions 3, 5, and 7 are shown in Figure 5a, while positions 9 and 10 are shown in Figures 5b, 5c.

1000 MUs were delivered for each measurement position, and absolute exposure in units of milliRoentgen (mR) was measured with the FLUKE BIOMEDICAL survey meter. The absolute dose in centiGray (cGy) was measured with the RadCal ionization chamber. The respective distance from the beam CAX was recorded, and the distance from the X-ray source was calculated based on the known geometry of the ZAP-X of SAD=45cm. For each measurement station, three readings were acquired. For standardization purposes, the readings were adjusted where necessary by an inverse square correction factor for equivalence at a 1m distance from the beam CAX. Lastly, the readings were expressed as a percentage of the reference dose delivered. Table 1 shows exposure measurements, and Table 2 shows dose measurement-based determination of X-ray Leakage Radiation. The position of the measured points and their distance from CAX was dictated by the inside dimensions of the ZAP-X treatment capsule in the patient and target planes. Measurement point distances for the survey meter are 2.8 cm smaller than for the RadCal ionization chamber, as both detectors were physically mounted on the inside surface of the treatment capsule; the center of the detection volume for the bulky survey meter is 2.8 cm closer to the CAX than the center of the flat pancake detector.

**Table 1:** Measurement stations for Exposure readings, distance from CAX, exposure, distance from the source, inverse square correction, adjusted exposure, dose to water, and percent leakage Three exposures were taken for each measurement point, with the mean value recorded in the table. The dose to water was calculated by multiplying the exposure by 0.97cGy/R for 3MV photon radiation. X: Exposure; mR: milliRoentgen; ISC: Inverse Square Correction; D: Dose

Station	Distance to CAX [cm]	X [mR]	Distance to Source [cm]	ISC	Adjusted mR	D Water [cGy]	Percentage Leakage [%]
1	61	10.65	75.80	0.478	5.09	0.00494	0.000494
2	50	13.1	67.27	0.376	4.93	0.00478	0.000478
3	46	14.6	64.35	0.344	5.03	0.00488	0.000488
4	100	6.15	109.7	1.00	6.15	0.00597	0.000597
5	76	7.00	88.32	0.649	4.54	0.00440	0.000440
6	55	16.4	71.06	0.420	6.89	0.00668	0.000668
7	33	17.8	55.80	0.259	4.61	0.00447	0.000447
8	38	13.3	58.90	0.288	3.84	0.00372	0.000372
9	65	17.5	65.00	0.423	7.39	0.00717	0.000717
10	65	16.2	65.00	0.423	6.82	0.00662	0.000662
11	70	13.9	70.00	0.490	6.81	0.00661	0.000661
12	59	18.0	59.00	0.348	6.27	0.00608	0.000608
13	70	17.25	70.00	0.490	8.45	0.00820	0.000820

**Table 2:** Measurement stations for dose measurements, distance from CAX, Dose reading, distance from the source, inverse square correction, adjusted dose, percentage leakage radiation Three dose measurements were taken for each measurement point, with the mean value recorded in the table. D: Dose; ISC: Inverse Square Correction

Station	Distance to CAX [cm]	D [µSv]	Distance to Source [cm]	ISC	Adjusted D µSv	D [cGy]	Percentage Leakage [%]
1	63.8	95.21	78.07	0.507	48.27	0.00483	0.000483
2	52.8	128.0	69.37	0.400	51.21	0.00512	0.000512
3	48.8	269.0	66.38	0.366	98.59	0.00986	0.000986
4	102.8	66.95	112.2	1.047	70.11	0.00701	0.000701
5	78.8	70.4	90.74	0.685	48.21	0.00482	0.000482
6	57.8	110.8	73.25	0.446	49.44	0.00494	0.000494
7	35.8	176.0	57.50	0.275	48.38	0.00484	0.000484
8	40.8	118.5	60.74	0.307	36.36	0.00364	0.000364
9	67.8	144.0	67.80	0.460	66.20	0.00662	0.000662
10	67.8	173.6	67.80	0.460	79.82	0.00798	0.000798
11	72.8	171.1	72.80	0.530	90.68	0.00907	0.000907
12	61.8	219.9	61.80	0.382	83.99	0.00840	0.000840
13	72.8	99.0	72.80	0.530	52.47	0.00525	0.000525

D Water in Table 1 was calculated by applying the f factor for water for the 3 MV energy range by applying the formula:

f_med_ = 0.876 x (µ_en_/ρ)_med_/(µ_en_/ρ)_air_ = 0.97.

The analysis of X-ray Target leakage for exposure-based measurements resulted in 0.000820%, while for dose-based measurements resulted in 0.000986%. Both values are below 0.001%.

## Discussion

As the X-ray target shielding of the ZAP-X collimator housing consists of 5.0 TVL of Tungsten, the expected dose rate at 1m from the target is expected to be 0.001 % of the primary reference dose defined at the isocenter. The radiation leakage measurements showed a maximum value of 0.000986 % in the patient plane and 0.000907% in the target plane, below the expected level of 0.001 %. The results confirm the correct implementation of the ZAP-X shielding design requirements and demonstrate its radiation leakage per unit dose delivered to be a factor of up to 51 smaller compared to a typical value of 0.05 % derived from the IEC standard.

As the IEC 60601-2-1 standard is used for the design of typical Radiotherapy Linear Accelerators (Linacs), conventional multi-purpose and dedicated Linacs with Multi-leaf collimators (MLC) do not exceed 0.05 % of leakage radiation [3]. The typical IMRT factors for MLC-defined treatments are 3-4 so that for a single fraction treatment to 20 Gy, 6000 MUs to 8000 MUs will be delivered. The ZAP-X uses approximately 12,000 MUs for complex treatment of 20 Gy with an IMRT factor of 6 [4]. Since the X-ray target leakage is expressed as a percentage of reference dose delivered by each beam throughout the treatment, the total ZAP-X radiation leakage for a 25mm reference collimator is expected to be 0.000986 % x 12,000 cGy = 0.118 cGy, which is equivalent to 1.18 mSv (for a Quality Factor of 1). A conventional Linac is expected to exert a target leakage radiation intensity of 0.05 % x 6000 cGy = 3.0 cGy, equivalent to 30 mSv. Even though the ZAP-X delivers approximately twice the amount of MUs, the total ZAP-X target radiation leakage is a factor of 25.4 lower compared to conventional Linacs. The reason for such a low ZAP-X leakage radiation can be found in the system's more compact size and lower beam energy, which results in smaller TVL thickness and thereby provides more shielding effect for an equivalent amount of shielding material.

General Radiation Shielding guidelines are published as consensus by the National Council on Radiation Protection (NCRP) report no. 151 for Radiotherapy operations using Linacs and specialized Radiosurgery devices [5]. When the ZAP-X was designed, these guidelines were strictly followed by providing the shielding materials as integrated system components in the axial and oblique shells of the gyroscopic ZAP-X skeleton [6]. The shielding effect of the ZAP-X ensures extremely low radiation leakage within the treatment room, allowing members of the public to access the room during radiation delivery. This same philosophy of ultra-low radiation exposure was applied to the X-ray target shielding that provides for a very low patient whole body dose which was investigated here. Both persons close to the treatment system within the treatment room and the patient herself are expected to receive a small fraction of approximately 10% compared to conventional Radiotherapy operations.

## Conclusions

In a side-by-side comparison of the X-ray target leakage radiation of the ZAP-X with conventional Linear Accelerators, the ZAP-X produces a significantly lower dose received by the patient during the treatment delivery. The ZAP-X's target leakage per beam reference dose is 51 lower than the expected standard; during a typical treatment, the target leakage radiation is 25.4 lower compared to this standard. The Tungsten Shielding thickness alongside the ZAP-X Linear Accelerator consists of 10.4cm or 3.5 TVL. However, only oblique beams from the X-ray target reach the patient during the treatment delivery, penetrating the tungsten shield in directions that result in a minimum thickness of 5.0 TVL.

The secondary cancer incidence and mortality rates induced by whole-body radiation during treatment are directly proportional to the amount of radiation; therefore, such side effects are expected to be lower by a factor of 25.4 for ZAP-X treatments compared to the expected Linac standard. The ZAP-X target shielding is significantly superior to that of conventional Linear Accelerators. Therefore, the ZAP-X can be considered a safe alternative for patient treatments with a significantly lower risk of patient morbidity and mortality than conventional Linear Accelerators.
